# *Anaplasma* spp. in domestic ruminants: evidence of circulation and molecular analysis of *Anaplasma phagocytophilum* and *Anaplasma capra* in Slovakia

**DOI:** 10.3389/fcimb.2026.1890232

**Published:** 2026-07-15

**Authors:** Maroš Kostičák, Monika Drážovská, Božena Kočíková, Jakub Lipinský, Andrea Pelegrinová, Patrícia Petroušková, Anna Ondrejková, Ľuboš Korytár, Boris Vojtek, Katarína Franková, Jana Mojžišová Vaščinec, Kristína Hudáková, Marián Prokeš

**Affiliations:** Department of Epizootiology, Parasitology and Protection of One Health, University of Veterinary Medicine and Pharmacy in Košice, Košice, Slovakia

**Keywords:** *Anaplasma capra*, *Anaplasma phagocytophilum*, cattle, goat, one health, sheep, Slovakia

## Abstract

The geographic expansion of ticks in Europe has increased exposure to tick-borne pathogens, particularly *Anaplasma phagocytophilum*, the causative agent of anaplasmosis in humans and animals. The recently described *A. capra* has raised interest as an emerging tick-borne agent with potential veterinary and public health relevance. This study aimed primarily to assess the molecular occurrence of *A. phagocytophilum* in domestic ruminants and secondarily to screen for the presence of *A. capra* in Slovakia. From 2023 to 2025, 466 blood samples were collected from sheep (n = 183), goats (n = 118), and cattle (n = 165) from farms in Slovakia. Samples were tested by nested PCR targeting the *16S rRNA* gene of *Anaplasma* spp. Subsequently, *Anaplasma*-positive samples were screened for *A. capra* using a *groEL* gene-based assay. All *Anaplasma*-positive amplicons were confirmed by Sanger sequencing. No *Anaplasma* DNA was detected in cattle, whereas 15 sheep (8.2%) and 13 goats (11.0%) were PCR-positive for *Anaplasma* spp. Sequencing and phylogenetic analysis confirmed all positive samples as *A. phagocytophilum*, with 99.08-100% identity to reference sequences. Comparative analysis of the partial *16S rRNA* fragment revealed nine sequence variants among domestic ruminants, including a dominant variant shared by sheep and goats. *A. capra* DNA was not detected in any screened sample. The detection of *A. phagocytophilum* DNA in sheep and goats provides baseline molecular evidence of its occurrence in small ruminants in Slovakia. However, because only a partial *16S rRNA* fragment was analysed and no tick samples were included, conclusions regarding strain-level diversity, local transmission cycles, and zoonotic relevance should be interpreted cautiously.

## 1 Introduction

The epidemiology of tick-borne diseases (TBDs) in Central Europe is changing in response to environmental, ecological, and anthropogenic factors. Anthropogenic climate change has led to a northward and altitudinal expansion of *Ixodes ricinus*, the primary vector for numerous bacterial agents (Stanko et al., 2022).

Within the family Anaplasmataceae, the genus *Anaplasma* consists of obligate intracellular bacteria that infect a broad spectrum of vertebrate hosts, including humans (Dumler et al., 2001; Karlsen et al., 2020).

*Anaplasma phagocytophilum* is a host generalist pathogen causing tick-borne fever (TBF) in ruminants and human granulocytic anaplasmosis (HGA) (Stuen et al., 2013). In domestic ruminants, it targets neutrophil granulocytes, leading to hyperthermia, decreased milk yield, and significant immunosuppression. This immunomodulatory effect renders animals susceptible to fatal secondary infections, such as pneumonic pasteurellosis, causing substantial economic losses (Woldehiwet, 2006).

The recently described *A. capra* has further complicated the zoonotic landscape. First isolated from asymptomatic goats in China in 2012 and later identified as a human pathogen in 2015, it has since been reported in various mammalian hosts across Asia and sporadically in Europe (Li et al., 2015; Altay et al., 2024). Its ability to cause febrile illness in humans highlights the need for molecular screening in livestock that act as bridge hosts between wildlife reservoirs and human populations (Jouglin et al., 2019; Peng et al., 2021).

In Slovakia, despite high seroprevalence rates reported in previous years, molecular data confirming the current infection pressure and identifying circulating species remain limited (Derdáková et al., 2011; Kostičák et al., 2025).

This study aimed to investigate the molecular occurrence of *Anaplasma* spp. in domestic ruminants in Slovakia, with emphasis on *A. phagocytophilum* circulation and screening for *A. capra*.

## 2 Materials and methods

### 2.1 Study localities and sample collection

**Figure 1:**
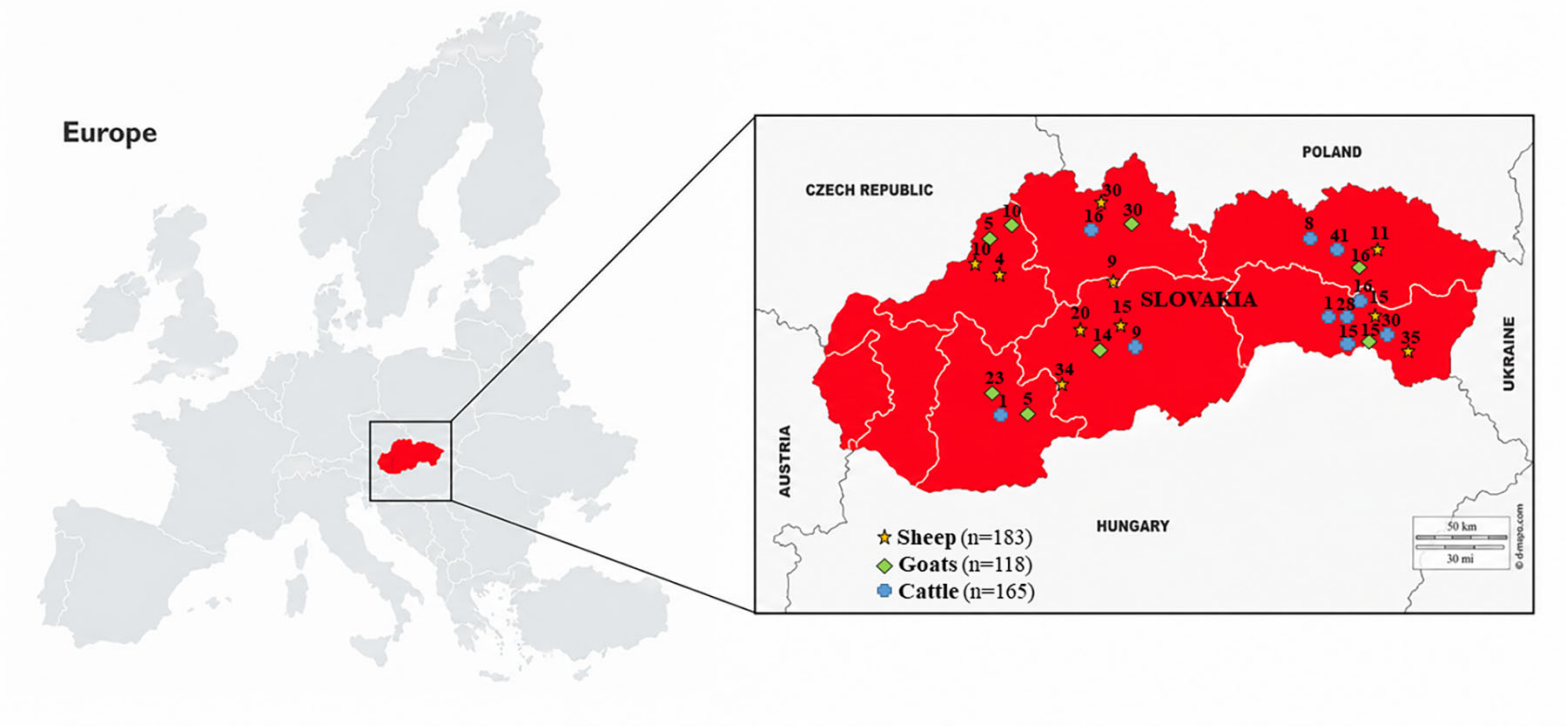
Sampling localities and number of samples from sheep, goats and cattle.

Blood samples were collected between 2023 and 2025 during spring and autumn. A total of 466 blood samples were collected from sheep (n = 183), goats (n = 118), and cattle (n = 165) originating from multiple family and commercial farms in different regions of Slovakia (**Figure 1**; **Table 1**). The selection of sampling sites was intended to cover geographically and ecologically diverse environments, including lowland, hilly, and mountain or submountain areas with different altitudes, climatic conditions, and expected tick exposure. Animals were kept under pasture-based conditions or had regular outdoor access. All sampled animals were females from milk-producing herds; therefore, sex and production type were not evaluated as explanatory variables in the statistical analysis. Biological material was collected from randomly selected animals. The selected individuals had no pathological findings and no clinical symptoms of a transmissible infectious disease at the time of sampling. Before sampling, the general clinical condition of each animal was assessed. Blood was collected with the consent of the owners or keepers by a veterinarian. All procedures were carried out in accordance with good veterinary practice, with emphasis on animal welfare, minimization of stress, and personnel safety. Blood collection was performed under aseptic conditions using sterile disposable material. Whole blood intended for molecular analysis was collected into EDTA tubes and stored at −20 °C until laboratory examination.

**Table 1:** Sampling localities, number of domestic ruminants and PCR detection of *Anaplasma* spp.

Locality / area	Approx. altitude (m a.s.l.)	Sheep			Goats			Cattle		
		n	PCR		n	PCR		n	PCR	
									+	%
			+	%		+	%			
Košické Olšany	~230	15	0	0.0	—			—		
Sedliacká Dubová	~520	30	6	20.0	30	9	30.0	—		
Zvolen	~380	34	8	23.5	—			—		
Brezno+Polhora+Hronec	~487	15	0	0.0	—			—		
Demjata	~310	11	0	0.0	—			—		
Liptovský Mikuláš	~577	9	0	0.0	—			—		
Chvatimech	~460	20	0	0.0	—			—		
Papradno	~400	10	0	0.0	—			—		
Udiča	~360	4	0	0.0	—			—		
Čalovka	~150	35	1	2.8	—			—		
Slanská Huta	~430	—			15	0	0.0	—		
Zlaté Moravce	~200	—			23	3	13.0	1	0	0.0
Jabloňovce	~190	—			5	0	0.0	—		
Brezno+Rohozná	~487	—			14	0	0.0	—		
Fintice	~250	—			16	0	0.0	—		
Dolný Hričov	~340	—			10	1	10.0	—		
Klieština	~420	—			5	0	0.0	—		
Tulčík	~375	—			—			41	0	0.0
Ďačov	~430	—			—			8	0	0.0
Zemplínska Teplica	~108	—			—			30	0	0.0
Bohdanovce	~185	—			—			15	0	0.0
Čižatice	~210	—			—			16	0	0.0
Nižný Klátov	~335	—			—			1	0	0.0
Ruskov	~190	—			—			28	0	0.0
Brezno+Lúčky	~488	—			—			9	0	0.0
Dolný Kubín	~500	—			—			16	0	0.0
**Total**		**183**	**15**	**8.20**	**118**	**13**	**11.02**	**165**	**0**	**0.0**

### 2.2 DNA extraction and molecular analysis

Genomic DNA was isolated from whole blood using the DNeasy Blood & Tissue Kit (QIAGEN, Hilden, Germany) following the manufacturer’s protocol. After extraction, DNA concentration and purity were assessed for all samples using NanoDrop spectrometry (Thermo Fisher Scientific, Waltham, MA, USA) before PCR analysis. All extracted DNA samples contained sufficient measurable DNA for downstream molecular testing. Although DNA quantity and purity were assessed spectrophotometrically, no host housekeeping gene PCR or endogenous internal amplification control was included in the diagnostic workflow; therefore, PCR inhibition or reduced DNA amplifiability could not be assessed individually for each sample. All samples were investigated for *Anaplasma* spp. by nested PCR targeting *16S rRNA* gene using specific primers pairs ACn-16S-F1 (5′-CACATGCAAGTCGAACGGATTATTC-3′) and ACn-16S-R1 (5′-TTCCGTTAAGAAGGATCTAATCTCC-3′) and ACn-16S-F2 (5′-AACGGATTATTCTTTA TAGCTTGCT-3′) and ACn-16S-R2 (5′-GGCAGTATTAAAAGCAGCTCCAGG-3′). The expected size of products was 932 bp for the first round of PCR and 546 bp for the nested PCR. Both PCR reactions were performed in a final volume of 25 µL. The reaction mixture consisted of 12.5 µL 2 x PPP Master Mix (Top-Bio, Vestec, Czech Republic), 1.0 µL of each primer (30 M), 9.5 µL PCR-grade water, and 1.0 µL of template. The both amplification reactions were performed under the following conditions: initial denaturation at 94 °C for 2 min, followed by 40 or 30 cycles of denaturation at 94 °C for 30 s, annealing at 55 °C for 30 s, and extension at 72 °C for 60 s for the first and the second round, respectively, with a final elongation at 72 °C for 5 min. DNA previously extracted in our laboratory and confirmed by sequencing (unpublished data) was used as a positive control, whereas PCR-grade water was used as a negative control.

*Anaplasma* spp. positive samples were also screened for *A. capra* by conventional PCR, using specific primers (GroEL-F: 5′-GAAGAGCATCAAACCCGAAG-3′ and GroEL-R: 5′-CTGCTCGTG ATGCTATCGG-3′) to amplify 874 bp fragment of *groEL* gene. PCR reaction mixture was prepared as described above. Amplification was performed under following conditions: initial denaturation at 94 °C for 2 min, followed by 40 cycles of denaturation at 94 °C for 30 s, annealing at 55 °C for 30 s, and extension at 72 °C for 60 s, with a final elongation at 72 °C for 5 min. PCR amplicons were visualized on 1.0% agarose gel with a GelRed Nucleic Acid Stain (Biotium, Fremont, CA, USA). A confirmed *A. capra*-positive control was not available for the *groEL*-based assay. Standard precautions to reduce carry-over contamination were applied during PCR processing, including separated pre-PCR and post-PCR handling steps, use of sterile disposable materials and PCR-grade reagents, inclusion of no-template negative controls, and sequencing confirmation of all *Anaplasma* spp. *16S rRNA*-positive amplicons.

### 2.3 DNA sequencing and phylogenetic analysis

Purification and Sanger sequencing of all PCR products were performed by Microsynth (Balgach, Switzerland). Acquired sequences were compared to reference sequences deposited in the GenBank database using BLASTn (https://blast.ncbi.nlm.nih.gov/Blast.cgi; accessed on 19 March 2026) and then submitted to the GenBank database using the BankIt submission portal (https://www.ncbi.nlm.nih.gov/WebSub/; accessed on 1 April 2026) under accession numbers PZ231792–PZ231819.

Phylogenetic analysis was performed on the basis of partial *16S rRNA* sequences (546 bp) generated in this study together with selected homologous reference sequences retrieved from GenBank. Multiple-sequence alignment was carried out in MEGA version 12.1 (Molecular Evolutionary Genetics Analysis, Temple University, Philadelphia, PA, USA) (Kumar et al., 2024) using the ClustalW algorithm and subsequently inspected visually. The phylogenetic tree was constructed using the Maximum Likelihood method. The best-fitting nucleotide substitution model was identified as Kimura 2-parameter with invariant sites (K2+I). The robustness of the resulting topology was evaluated by bootstrap analysis with 1, 000 replicates.

To assess sequence variation within the analysed fragment, the obtained sequences were analysed and compared with the reference sequence (CP006616: complete genome of *A. phagocytophilum* strain HZ2) and with each other in Geneious Prime v2025.1.3 (Biomatters Ltd., Auckland, New Zealand).

### 2.4 Statistical analysis

Apparent molecular prevalence was calculated as the proportion of PCR-positive animals among tested animals. Ninety-five percent confidence intervals were estimated using the Wilson score method. Differences in PCR positivity among host species were evaluated using the chi-square test for the overall comparison and Fisher’s exact test for pairwise comparisons where appropriate. Statistical significance was set at p < 0.05. Because all sampled animals were females from milk-producing herds, sex and production type were not included as explanatory variables. Multivariable logistic regression was not performed because of the low number of PCR-positive animals, the absence of PCR-positive cattle, unequal sample sizes across localities, and incomplete individual-level metadata for variables such as exact age, tick infestation history, and detailed grazing-management characteristics. Therefore, the statistical analysis was focused on host-species comparisons and descriptive locality-level interpretation.

## 3 Results

The molecular survey revealed an overall apparent *Anaplasma* spp. prevalence of 6.0% (28/466; 95% CI: 4.2-8.5) in Slovak domestic ruminants (**Table 2**). *Anaplasma* spp. DNA was detected in 15/183 sheep (8.2%; 95% CI: 5.0-13.1) and 13/118 goats (11.0%; 95% CI: 6.6-17.9), whereas no *Anaplasma* DNA was identified in any of the 165 cattle samples (0.0%; 95% CI: 0.0-2.3). *A. capra* DNA was not detected in any *Anaplasma* spp.-positive sample screened using the *groEL*-based assay. PCR positivity differed significantly among host species (chi-square statistic = 17.34, df = 2, p = 0.00017). Pairwise comparisons using Fisher’s exact test showed no significant difference between sheep and goats (p = 0.423). In contrast, PCR positivity differed significantly between sheep and cattle (p = 5.87 x 10^-5), goats and cattle (p = 7.67 x 10^-6), and small ruminants combined and cattle (p = 4.92 x 10^-6).

**Table 2:** Molecular detection of *Anaplasma* spp. in domestic ruminants.

Host species	n	*16S rRNA* positive, n	Apparent prevalence (%)	95% CI	*A. capra groEL* PCR among 16S-positive samples
Cattle	165	0	0.0	0.0-2.3	Negative
Sheep	183	15	8.2	5.0-13.1	Negative
Goats	118	13	11.0	6.6-17.9	Negative
Total	466	28	6.0	4.2-8.5	Negative

**Figure 2:**
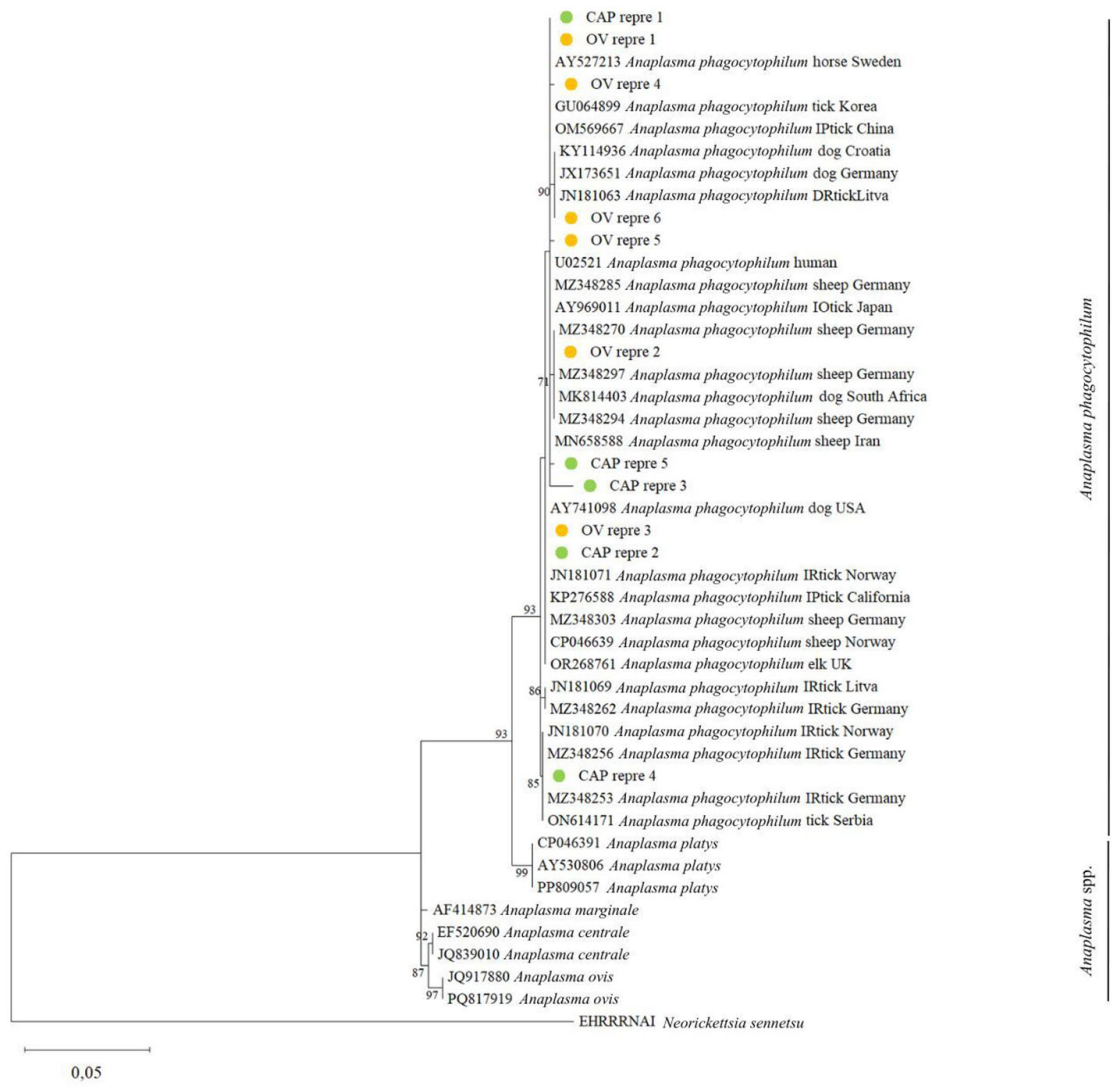
Phylogenetic tree of the partial *16S rRNA* gene (546 bp) of *A. phagocytophilum*, constructed by the Maximum Likelihood method using the Kimura 2-parameter model with the proportion of invariant sites (K2+I). Bootstrap analysis values ​​(1000 replications) higher than 70 are shown at the branch point. Representative sequences obtained in this study are highlighted with a dot (orange for sheep; green for goats). For *A. phagocytophilum* sequences, hosts and country are also indicated. The phylogenetic tree was rooted using the outgroup *Neorickettsia sennetsu*.

Phylogenetic analysis demonstrated that all sequences obtained in this study clustered within the *A. phagocytophilum* group (**Figure 2**) and showed a high level of nucleotide identity (99.08–100%) with reference sequences (listed in **Supplementary Table 1**).

Comparative analysis of the partial *16S rRNA* fragment revealed sequence heterogeneity among the ruminant-derived isolates. Of the total number of 15 sequences obtained from sheep, six representative sequences were determined (**Supplementary Figure 1**). Six sequences (OV repre 1: PZ231798, PZ231800 - PZ231802, PZ231804, PZ231805) were 100% identical to the reference sequence. In the next nine sequences, point mutations A29G (OV repre 2: PZ231792 and PZ231794), G36A (OV repre 3: PZ231793 and PZ231795 - PZ231797), T111C (OV repre 5: PZ231803), G328A (OV repre 6: PZ231806) and T498A (OV repre 4: PZ231799) were confirmed.

Among the 13 caprine sequences, five representative sequence variants were identified (**Supplementary Figure 2**). Eight goat-derived sequences (CAP repre 1: PZ231807, PZ231809, PZ231810, PZ231812, PZ231815–PZ231817, PZ231819) were 100% identical to the reference sequence. One sequence (CAP repre 2: PZ231808) contained a substitution G36A. CAP repre 3 (PZ231811) carried five nucleotide substitutions, namely A54T, G55A, G66A, A68C and G77A. Two substitutions A28G and G36A were identified for CAP repre 4 (PZ231813 and PZ231814) sequence. Finally, CAP repre 5 (PZ231818) contained a substitution T173C.

Collapsing identical sequence types detected in sheep and goats resulted in the identification of nine sequence variants within the analysed 546 bp *16S rRNA* fragment (**Supplementary Table 1**). The dominant variant was detected in both sheep and goats, whereas less abundant variants showed host-restricted distribution within the analysed cohort. Two ruminant-associated sequence variants were identical within this partial *16S rRNA* fragment to human-derived sequences available in GenBank; however, this finding should be interpreted as sequence similarity within a conserved marker and not as evidence of direct zoonotic transmission.

At the locality level, PCR-positive small ruminants were detected only in selected areas. In sheep, PCR-positive animals were detected in Sedliacká Dubová, Zvolen, and Čalovka, whereas in goats, positive animals were detected in Sedliacká Dubová, Zlaté Moravce, and Dolný Hričov. Because of unequal sample sizes across localities, non-uniform representation of host species, absence of PCR-positive cattle, and the descriptive sampling design, formal statistical modelling of prevalence by region or altitude was not performed.

## 4 Discussion

Molecular screening of Slovak domestic ruminants revealed a host-associated pattern of *Anaplasma* spp. detection within the examined cohort. *A. phagocytophilum* DNA was detected in small ruminants: 15 of 183 sheep (8.2%) and 13 of 118 goats (11.0%) were PCR-positive. In contrast, no *Anaplasma* DNA was detected in any of the 165 cattle samples. Screening for *A. capra* did not detect *A. capra* DNA among the *Anaplasma* spp.-positive samples screened with the *groEL*-based assay. These findings provide baseline molecular evidence of *A. phagocytophilum* occurrence in Slovak sheep and goats, while the negative findings in cattle and the *A. capra* screening should be interpreted cautiously within the limitations of the study design.

The PCR positivity observed in sheep and goats supports the molecular occurrence of *A. phagocytophilum* in Slovak small ruminants. Earlier molecular data from Slovakia detected *A. phagocytophilum* in sheep flocks with prevalence values ranging from 0.9% to 5.7%, indicating that this pathogen had already been detected in local small ruminant populations (Derdáková et al., 2011). The higher prevalence recorded in the present study may reflect differences in sampling period, local tick exposure, pasture management, ecological conditions, or the selection of farms included in the survey. Similar variability has been reported in European studies, where molecular prevalence differed according to geographic region, flock health status, vector exposure, and diagnostic approach (Bauer et al., 2021; Lysholm et al., 2023). The detection of PCR-positive small ruminants only in selected localities supports a patchy pattern of *A. phagocytophilum* molecular occurrence, which is consistent with the ecology of tick-borne pathogens maintained in local tick-host-environment systems (Stuen et al., 2013; Stanko et al., 2022).

Goats showed slightly higher PCR positivity than sheep among the examined species. The detection of *A. phagocytophilum* DNA in 11.0% of goats is epidemiologically relevant from a surveillance perspective and may be related to pasture-based management and regular outdoor access, which increase the probability of contact with tick habitats. In Sweden, *A. phagocytophilum* DNA was detected in goats at a lower molecular prevalence, while exposure to *Anaplasma* spp. was more broadly documented by serological testing (Lysholm et al., 2023). However, direct comparison between studies is limited by differences in sampling strategy, season, diagnostic methods, herd management, local tick abundance, and ecological conditions. Without longitudinal sampling and parallel testing of ticks from the same farms, it is not possible to determine whether goats act only as repeatedly exposed hosts or contribute more actively to local transmission.

The absence of PCR-positive cattle contrasts with findings from several regions where bovine infection with *A. phagocytophilum* has been documented. A recent global meta-analysis estimated the mean prevalence of *A. phagocytophilum* in cattle at 8.5%, although with considerable heterogeneity between countries and study designs (Abdoli et al., 2025). In our study, no *Anaplasma* DNA was detected in 165 cattle samples. This result may reflect lower current molecular detection in the sampled herds, differences in management and pasture exposure compared with small ruminants, or the difficulty of detecting transient bacteremia in peripheral blood. Therefore, the negative bovine results should be interpreted as non-detection of *Anaplasma* DNA in the analysed cohort, rather than as evidence that cattle are not exposed to *A. phagocytophilum* in Slovakia.

The non-detection of *A. capra* in the analysed samples is also noteworthy. *A. capra* has been recognized as an emerging zoonotic *Anaplasma* species and has been reported from domestic and wild ruminants, ticks, and humans (Li et al., 2015; Jouglin et al., 2019; Peng et al., 2021; Altay et al., 2024). In a large study from China, *A. capra* DNA was detected in 129 of 1, 453 blood samples from sheep and goats, corresponding to an overall prevalence of 8.9%, with positivity of 9.4% in goats and 7.8% in sheep (Peng et al., 2018). This study also identified genetic variability based on *gltA*, *groEL*, and *msp4* analyses, supporting the importance of small ruminants in molecular surveillance of this emerging pathogen. In contrast, no *groEL*-positive *A. capra* sample was detected in the present Slovak cohort. However, a confirmed *A. capra*-positive control was not available in our laboratory, and therefore the sensitivity of the assay under local laboratory conditions could not be fully verified. Consequently, this result should be interpreted strictly as non-detection of *A. capra* DNA in the screened samples, not as definitive evidence that *A. capra* is absent from Slovakia. Further screening of domestic ruminants, wildlife, and ticks will be necessary to assess whether this zoonotic species circulates in Slovak ecosystems.

The molecular prevalence recorded in Slovak small ruminants fits within the broader pattern of regional heterogeneity reported for *Anaplasma* infections in sheep and goats. Direct comparison between studies is limited by differences in host species, target genes, sampling design, vector ecology, and the *Anaplasma* species investigated. Nevertheless, molecular studies in small ruminants consistently show the value of combining PCR with sequencing. Peng et al. (2018) used PCR and phylogenetic analysis to demonstrate the occurrence and diversity of *A. capra* in sheep and goats in China. Similarly, Naeem et al. (2023) reported molecular detection and risk-factor analysis of *A. ovis* in sheep from Pakistan. Although *A. ovis* differs biologically from *A. phagocytophilum*, these studies illustrate that molecular surveillance is most informative when combined with host-level, farm-level, and vector-related data.

An important aspect of the present work is the sequence-based confirmation of all positive amplicons. All 28 PCR-positive samples were confirmed as *A. phagocytophilum* and deposited in GenBank. Analysis of the partial *16S rRNA* fragment identified six representative sequence variants in sheep and five in goats, which together formed nine sequence variants within the analysed marker. The dominant variant was shared by sheep and goats, whereas several less frequent variants were detected only in one host species. This finding indicates sequence variation among Slovak small-ruminant *A. phagocytophilum* samples, but it should not be interpreted as definitive strain-level differentiation.

The detected sequence variation is consistent with previous studies showing that *A. phagocytophilum* is a genetically heterogeneous species complex. Variability has been described both in conserved and more variable genetic loci, and different variants may circulate among different hosts within the same ecological area (Massung et al., 2002; Rar et al., 2011; Silaghi et al., 2011; Miranda et al., 2021; Myczka et al., 2024). Several Slovak sequences showed high similarity or identity with sequences previously reported from humans, domestic animals, wildlife, and ticks from different geographic regions, as summarized in **Supplementary Table 1**. This observation is epidemiologically relevant from a surveillance perspective. However, identity within a partial *16S rRNA* fragment indicates phylogenetic similarity only and does not prove host adaptation, pathogenicity, direct zoonotic transmission, or epidemiological linkage between domestic ruminants and humans. Higher-resolution approaches, including additional genetic markers such as *groEL*, *gltA*, *msp4*, *ankA*, and *msp2*, multilocus sequence typing, or whole-genome sequencing, would be needed to clarify the biological and epidemiological significance of these variants (Portillo et al., 2011; Miranda et al., 2021; Myczka et al., 2024).

Several limitations of our study should be acknowledged. The selection of farms depended partly on owner cooperation and logistical feasibility, which may have introduced sampling bias, geographical bias, management-related bias, and herd-level clustering. Individual-level data, including exact age, clinical status, tick infestation history, and previous treatment, were not consistently available, limiting the evaluation of risk factors. All sampled animals were females from milk-producing herds; therefore, sex and production type could not be assessed as independent risk factors. In addition, PCR testing of peripheral blood detects only animals with circulating bacterial DNA at the time of sampling and may underestimate infection when bacteremia is transient or below the detection threshold. Analytical validation parameters of the PCR assays were not experimentally determined. DNA concentration and purity were assessed by NanoDrop spectrophotometry, but no host housekeeping gene PCR or endogenous internal amplification control was included; therefore, PCR inhibition or reduced DNA amplifiability cannot be completely excluded for individual PCR-negative samples. Finally, no questing or attached ticks were collected, and the present study cannot directly assess vector infection rates, local infection pressure, reservoir competence, or complete transmission cycles.

Overall, the results support the molecular occurrence of *A. phagocytophilum* in Slovak sheep and goats and show sequence variation within the analysed partial *16S rRNA* fragment. The absence of PCR-positive cattle and the non-detection of *A. capra* provide important baseline information but require confirmation in broader surveillance studies. Future work should include longitudinal sampling, testing of questing and attached ticks, wildlife screening, and analysis of additional genetic markers to better define the ecology and potential zoonotic relevance of *A. phagocytophilum* variants circulating in Slovakia.

Sequencing of all positive amplicons confirmed *A. phagocytophilum* and revealed sequence variation within the analysed partial *16S rRNA* fragment, with nine sequence variants identified among sheep and goats. The close similarity of several Slovak variants to sequences previously reported from humans, domestic animals, wildlife, and ticks highlights the epidemiological relevance of small ruminants in regional tick-borne pathogen surveillance. However, because partial *16S rRNA* sequences provide limited discriminatory resolution, further studies using additional genetic markers, multilocus sequence typing, or whole-genome approaches are needed to clarify the zoonotic relevance and transmission dynamics of circulating variants.

Overall, these findings provide baseline molecular data on *A. phagocytophilum* in Slovak domestic ruminants and support integrated One Health surveillance involving livestock, ticks, wildlife reservoirs, and environmental risk factors. The obtained results provide useful data for assessing tick-borne pathogen occurrence in livestock populations in Slovakia.

## Data Availability

The dataset presented in this study can be found in an online repository (https://www.ncbi.nlm.nih.gov/genbank/) under the accession numbers PZ231792-PZ231819. Further inquiries can be directed to the corresponding author.
