# Supplementary material for: Anaplasma spp. in domestic ruminants: evidence of circulation and molecular analysis of Anaplasma phagocytophilum and Anaplasma capra in Slovakia
Source: Front Cell Infect Microbiol. 2026 Jul 15;16:1890232. doi: 10.3389/fcimb.2026.1890232 (PMC13414138; doi:10.3389/fcimb.2026.1890232)

Supplementary Material

# Supplementary Tables

**Supplementary Table 1.** Variants of *A. phagocytophilum 16S rRNA* gene fragment and reference sequences from GenBank database. Sequences obtained in this study in bold.

| **Variant** | **No. GenBank sequence (this study)** | **Host / representative name** | **Sequences with the highest identity (host / country)** |
| --- | --- | --- | --- |
| 1 | **PZ231798,**  **PZ231800–PZ231802, PZ231804, PZ231805** | OV repre 1 | CP166491 (100%) / human / Slovenia; MZ348285 (100%) / sheep / Germany; OM569668 (100%) / tick / China |
|  | **PZ231807, PZ231809, PZ231810, PZ231812, PZ231815–PZ231817, PZ231819** | CAP repre1 |  |
| 2 | **PZ231793,**  **PZ231795–PZ231797** | OV repre 3 | M73220 (100%) / human / USA; MZ348303 (100%) / sheep / Germany; JN181071 (100%) / tick / Norway |
|  | **PZ231808** | CAP repre 2 |  |
| 3 | **PZ231799** | OV repre 4 | CP006617 (99.82%) / human / USA; AY969011 (99.82%) / tick / Japan; GU064899 (99.82%) / tick / Korea |
| 4 | **PZ231792, PZ231794** | OV repre 2 | MK514403 (100%) / dog / South Africa; MZ348270 (100%) / sheep / Germany |
| 5 | **PZ231803** | OV repre 5 | MN658588 (100%) / sheep / Iran; CP006617 (99.82%) / human / USA; OM569668 (99.82%) / tick / China |
| 6 | **PZ231806** | OV repre 6 | JX173651 (100%) / dog / Germany; KY114936 (100%) / dog / Croatia; JN181063 (100%) / tick / Lithuania |
| 7 | **PZ231811** | CAP repre 3 | MZ348285 (99.08%) / sheep / Germany; CP006617 (99.08%) / human / USA |
| 8 | **PZ231813, PZ231814** | CAP repre 4 | MZ348256 (100%) / tick / Germany; ON614171 (100%) / tick / Serbia; JN181070 (100%) / tick / Norway |
| 9 | **PZ231818** | CAP repre 5 | OM569667 (99.82%) / tick / China; GU064899 (99.82%) / tick / Korea |

# Supplementary Figures

**Supplementary Figure 1.** Aligment of five obtained representative ovine sequences (546 bp fragment of *16S rRNA* gene of *A. phagocytophilum*).


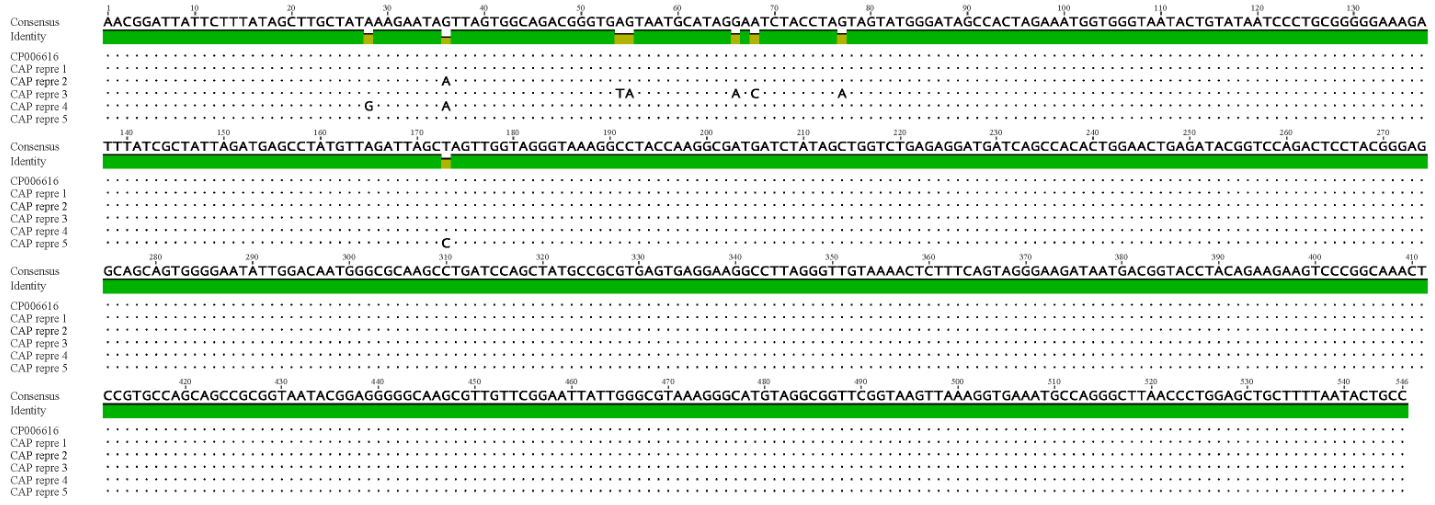


**Supplementary Figure 2.** Aligment of six obtained representative caprine sequences. (546 bp fragment of *16S rRNA* gene of *A. phagocytophilum*).


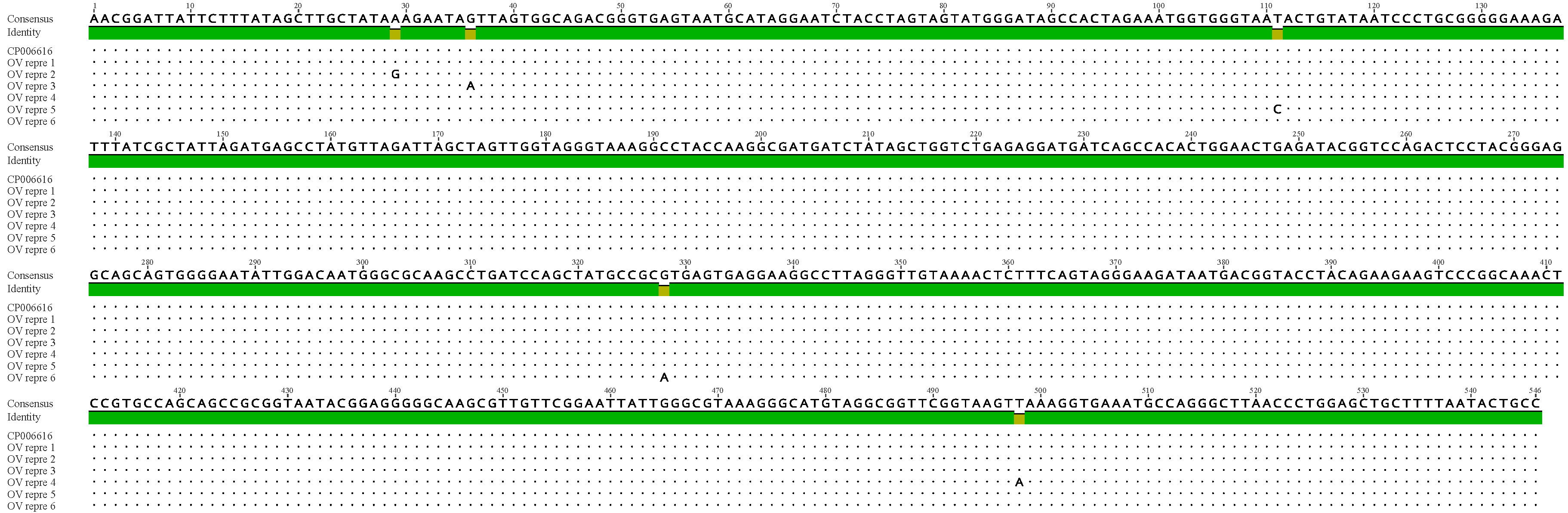

Supplement: Supplementary file 1 [file DataSheet1.docx]
